# Proteins Extraction and Characterization in Spirulina Biomass: A Comparative Study of High-Pressure Homogenization and Alkaline Methods

**DOI:** 10.3390/foods14223942

**Published:** 2025-11-18

**Authors:** Eleonora Muccio, Rossella Francesca Lanza, Francesco Marra, Donatella Albanese, Francesca Malvano

**Affiliations:** 1 Department of Industrial Engineering, University of Salerno, 84084 Fisciano, SA, Italy; emuccio@unisa.it (E.M.); rlanza@unisa.it (R.F.L.); fmarra@unisa.it (F.M.); fmalvano@unisa.it (F.M.); 2 Department of Agricultural, Forest and Food Sciences, University of Turin, 10124 Torino, TO, Italy

**Keywords:** Spirulina, protein extraction, protein-based gel, high-pressure homogenization (HPH)

## Abstract

The growing demand for sustainable proteins has driven interest in *Limnospira platensis* (Spirulina) due to its high protein content. However, the presence of the cell wall limits the availability and recovery of proteins within it. Conventional alkaline extraction is widely applied but often results in low yields and excessive solvent consumption. This study compares the efficiency and functional properties of Spirulina proteins extracted using an alkaline method and high-pressure homogenisation (HPH) at 20, 50, 80 and 100 MPa. Following isoelectric precipitation, proteins were collected in precipitate and supernatant fractions and characterized for yield, solubility, phycobiliproteins content, emulsifying and foaming properties, water– and oil–holding capacity, thermal stability and rheological behaviour. Microscopy confirmed progressive cell disruption with increasing homogenization pressures. HPH at 50 MPa increased protein extraction by 28% compared to alkaline extraction and significantly (*p* < 0.05) improved solubility, oil-holding capacity, foaming and emulsion properties. Phycobiliproteins, particularly C–phycocyanin, were more efficiently recovered in HPH supernatants, achieving a higher purity index than the alkaline method. Rheological analysis showed weak gel-like network formation, whereas excessive mechanical stress reduced functionality. Overall, HPH emerges as an interesting method for obtaining Spirulina proteins with enhanced technological properties; however, pressure optimisation is required to avoid denaturation and functionality loss.

## 1. Introduction

In response to global population growth, estimated to reach nearly 10 billion by 2050, global protein demand is expected to increase significantly from 202 to up to 360–1250 million tons. Moreover, the global protein market is evolving as consumers’ demand for plant-based protein grows [1,2]. Conventional protein production systems, based primary on animal and plant sources, are unlikely to meet this demand due to the competition for arable land, intensive feed crop requirements and high environmental impacts such as greenhouse gas (GHG) emissions and biodiversity loss [3,4]. Notably, the agri-food sector accounts for approximately 26% of global GHG emissions, highlighting the urgent need for sustainable dietary transitions and the development of alternative protein sources with lower ecological footprints [5].

European policies such as the Green Deal and the Farm to Fork Strategy aim to promote healthy and environmentally sustainable food systems, ensuring adequate nutrition while minimizing environmental impact [6]. In this context, plant-based proteins—especially those derived from legumes—have gained attention for their nitrogen-fixing capacity, low fertiliser requirements and promising contribution to GHG reduction targets [7]. However, to meet the future demand for proteins, crop production should increase by requiring more arable land, increased water use and, consequently, higher environmental impacts [8]. This complex scenario highlights the need to identify and develop sustainable protein alternatives. In particular, the blue–green spiral-shaped *Limnospira platensis* (commonly known as Spirulina), have emerged as a promising source of sustainable proteins for the development of innovative food products due to their non-competitive use of arable land, their capacity to grow in non-potable water and their adaptability to both open and closed cultivation systems [9].

The high protein content of Spirulina, which ranges from 50% to 70% on a dry matter basis (DB) [10,11], exceeds that of animal-based proteins such as chicken, pork and beef (approximately 20–24% DB) [12,13] and is also significantly higher than that of most conventional plant-based sources, such as whole soybeans (about 40% DB) and peas (20–25% DB) [12,14,15]. Moreover, it is rich in polyunsaturated fatty acids (Omega-3 fatty acids), vitamins (particularly B_12_ and A), minerals (iron, magnesium) and chlorophyll [16,17]. In addition, Spirulina is an excellent source of phycobiliproteins, which are classified into three main types: C–phycocyanin (C–PC), allophycocyanin (APC) and phycoerythrin (PE) [18]. Among these, the blue water-soluble C–PC accounts for the highest fraction, ranging from 10 to 20% DB. The increasing attention toward C–PC is mainly due to its well-characterized antioxidant, anti-inflammatory and anticancer properties [18,19].

However, the presence of the Spirulina cell wall limits the bioavailability of the protein fraction, since it constitutes a physical barrier to the valuable intracellular components. The cell wall of Spirulina consists of four main layers (L–I–L–IV), including an inner fibrillar β–1,2–glucan layer and a peptidoglycan layer providing structural rigidity. This multilayered network is externally covered by a sheath of acidic polysaccharides and glycoproteins, which limits solvent permeability and contributes to the high mechanical strength of the cell wall [20]. Conventional methods such as alkaline, salt and solvent-based extraction exhibit low extraction efficiency, a long processing time and excessive solvent usage, primarily due to the cell wall permeability [21]. The use of non-conventional techniques as ultrasounds [22], pulsed electric field [10] and microwaves [11] has been studied for protein extraction from the biomass of different *Limnospira* species. Recently, the emerging non-thermal mechanical technology high-pressure homogenization (HPH) demonstrated its potential to disrupt the algal cell wall effectively. During the process, the application of high pressure forces the fluid to pass through a narrow valve gap, where the resulting cavitation, shear forces and turbulence lead to a progressive loss of microbial cell wall integrity [23]. HPH has been widely investigated as a disruptive technology for enhancing the extraction and functionality of proteins from Spirulina and other microalgae species. Magpusao et al. [24] highlighted that HPH treatments (300–900 bar) effectively disintegrated Spirulina cells, leading to significant modifications in particle size and viscoelastic properties of the whole homogenized biomass.

Moreover, Giannoglou et al. [21] studied the combined effect of pressure (100–600 MPa) and pH on protein extraction kinetics from Spirulina biomass, showing that moderate pressures at near neutral pH maximized C–phycocyanin purity while minimizing protein denaturation. Shkolnikov Lozober et al. [25] applied HPH at 50 MPa as a pretreatment to improve the gelation and solubility properties of Spirulina protein concentrate (SPC). These findings support the relevance of high-pressure homogenization as a suitable method for the extractability and techno-functional improvement of Spirulina proteins. However, to the best of the authors’ knowledge, no study has compared the functional properties of protein extracts from Spirulina obtained by the conventional alkaline method and HPH at different pressures (20, 50, 80 and 100 MPa). This work aims to evaluate and compare the impact of two different protein extraction methods, the alkaline and HPH at 20, 50, 80 and 100 MPa, on the techno-functional properties of Spirulina proteins. Specific focus is given to protein yields, solubility, water- and oil-holding capacity, foaming and emulsifying properties as well as rheological and thermal behaviour. The obtained results could be used to identify the most suitable extraction conditions for obtaining Spirulina protein ingredients with enhanced techno-functional properties, suitable for the formulation of Spirulina–based food products.

## 2. Materials and Methods

### 2.1. Materials

The *Limnospira platensis* (Spirulina) was purchased from Sevenhills Wholefoods (Sheffield, UK) in dried powder. As stated by the producer, it is mainly composed of proteins, carbohydrate and fats of 65.3, 12.8 and 0.8 g/100 g dry matter, respectively. The PierceTM Bradford Protein Assay Kit was purchased from Thermo Fisher Scientific Inc. (Waltham, MA, USA) and was used to determine the soluble protein content. The Slide–A–Lyzer dyalisis flasks were purchased from Thermo Fisher Scientific Inc. (Waltham, MA, USA). All other chemicals were of analytical grade and purchased from Merck (Darmstadt, Germany).

### 2.2. Protein Extraction from Spirulina

#### 2.2.1. Conventional Alkaline Extraction Method

The alkaline extraction of the Spirulina proteins from the Spirulina biomass (SB) was performed according to Shkolnikov Lozober et al. [25], with minor modifications. SB powder 5% *w*/*v* was suspended in an alkaline solution at pH 10 with 0.5 M NaOH and stirred for 2 h at room temperature. The suspension was then centrifuged (SL 8 Small Benchtop, Thermo Fisher Scientific, Waltham, MA, USA) at 10,000× *g* for 30 min at 20 °C. The resulting precipitate was subjected to a second alkaline extraction under the same previous conditions. The collected supernatants were combined and acidified to pH 3, corresponding to the isoelectric point of Spirulina proteins [26], by adding 0.5 M HCl under gentle stirring. The mixture was stored at 4 °C overnight to allow for protein precipitation. The precipitated proteins were recovered by centrifugation at 10,000× *g* for 30 min at 20 °C. The resulting precipitate was resuspended, and the pH was adjusted to 7 using 0.5 M NaOH. Prior to freeze-drying, the suspension was dialyzed against deionized water for 24 h using dialysis flasks (cut off: 3.5 kDa) (Slide–A–Lyzer dyalisis, Thermofisher, USA) to remove residual salts and enhance protein purity. The soluble fraction (supernatant) remaining after isoelectric precipitation was also freeze-dried (Home Pro Freeze dryer, Harvest Right, Salt Lake, UT, USA) for subsequent analysis.

#### 2.2.2. High-Pressure Homogenization (HPH) Extraction

Protein extraction via HPH was carried out following the procedure described by Magpusao et al. [24], with slight modifications. SB was suspended in distilled water at a concentration of 5% *w*/*v* and subjected to a two-pass homogenization using PANDA Plus 2000 homogenizer (GEA Niro Soavi, Parma, Italy). Samples were processed at four different pressures: 20, 50, 80 and 100 MPa. The inlet temperature of SB suspension was kept below 10 °C, and the outlet temperature did not exceed 30 °C to prevent the thermal degradation of proteins. After homogenization, the SB suspension was centrifuged at 4000× *g* for 10 min at 20 °C to separate the cell debris from the aqueous soluble fractions. The resulting supernatant was collected and adjusted to pH 3 by dropwise addition of 0.5 M HCl under gentle stirring. The suspensions were stored overnight at 4 °C to guarantee the full protein precipitation and then centrifuged at 10,000× *g* for 30 min. The precipitates and supernatants were neutralized with 0.5 M NaOH and dialyzed against deionized water for 24 h using a 3.5 kDa molecular weight cut-off (MWCO) dialysis flask. Finally, both dialyzed precipitate and supernatants were freeze-dried (Home Pro Freeze dryer, Harvest Right, Salt Lake, UT, USA) until further use.

**Figure 1:**
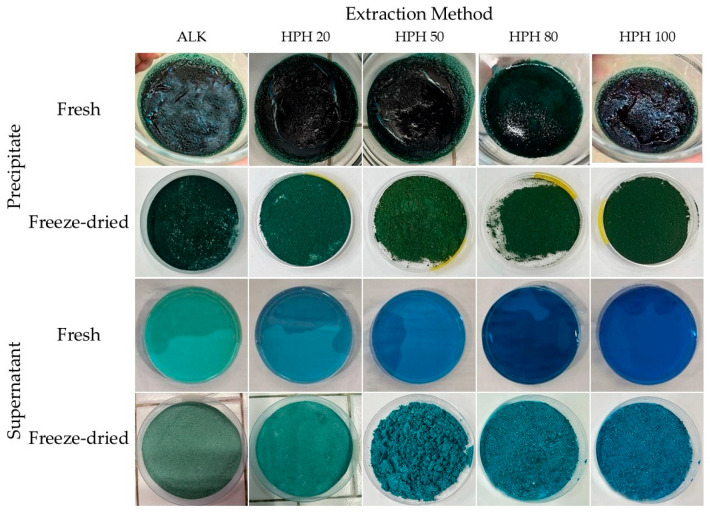
Visual comparison of precipitate and supernatant fractions obtained from Spirulina protein extraction using alkaline and high-pressure homogenization (20–100 MPa) methods, before and after freeze-drying.

All freeze-dried extracts were labelled according to Table 1, and the corresponding images are shown in Figure 1.

### 2.3. Optical Microscopy

The morphological evaluation of Spirulina cells was investigated by optical microscopy before and after alkaline and HPH treatments, following Magpusao et al. [24], with minor modifications. Aliquots of 100 μL for each suspension were placed on glass slides and covered with coverslips to avoid air bubble formation. Observations were performed using an optical microscope (Eclipse Ci-S microscope, Nikon Instruments Inc., Tokyo, Japan) coupled with a 5.0 MP camera (Moticam 580, Motic, Barcelona, Spain) and Motic Images Plus 2.0 software (Motic, Barcelona, Spain). Micrographs of all samples were acquired at 100× magnification.

### 2.4. Protein Content and Extraction Efficiency

Protein content and yield calculations were performed according to the method proposed by Purdi et al. [22], with minor modifications. Protein content was determined by the Kjeldahl method [27] by using a nitrogen-to-protein (NTP) conversion factor of 6.25, and values were expressed as grams of protein per 100 g of freeze-dried extract, according to the following Equation (1):

(1) $$Protein\;content\left(\%\right)=\frac{N_{tot}\;\cdot \;NTP}{m_{sample}}\;\cdot \;100$$

where $N_{tot}$ is the mass of nitrogen (g) in (-P) or (-S) and $m_{sample}$ is the mass of the freeze-dried extract (g) used for the analysis.

Protein yield was calculated as the mass of proteins ($P_{tot}$) in the precipitate and supernatant fractions against the initial proteins in Spirulina biomass ($P_{SB}$), using Equation (2):

(2) $$Protein\;yield\left(\%\right)=\frac{P_{tot}}{P_{SB}}\;\cdot \;100$$

Total protein extraction yield was calculated as the sum of the mass of proteins in the precipitates ($P_{tot-P}$) and supernatant ($P_{tot-S}$) for each extraction relative to the total protein content in Spirulina biomass ($P_{SB}$), according to Equation (3):

(3) $$Total\;protein\;extraction\;yield\left(\%\right)=\frac{P_{tot-P}+P_{tot-S}}{P_{SB}}\;\cdot \;100$$

### 2.5. Determination of Protein Solubility

Protein solubility was determined for the freeze-dried samples (ALK-P; ALK-S, HPH 20-S, HPH 50-S, HPH 80-S and HPH 100-S) according to Zhang et al. [28], with slight modifications. The ALK-P sample was suspended to 0.1% *w*/*v* in phosphate buffer solution (PBS) 0.1 M and the solubility was evaluated at different pH values ranging from 1 to 11 while the supernatant fractions (ALK-S, HPH 20-S, HPH 50-S, HPH 80-S, HPH 100-S) were suspended to 0.5% *w*/*v* and their solubility was evaluated at pH 7. Each suspension was magnetically stirred for 60 min at 20 °C and then centrifuged at 2000× *g* for 15 min at 20 °C. The resulting supernatants were collected for soluble protein analysis. The soluble protein concentration was determined using Bradford’s assay. Absorbance was measured at 595 nm using a VWR V-3000PC spectrophotometer (VWR International, Milano, Italy), and values were interpolated from a standard curve prepared with bovine serum albumin (BSA). Protein solubility was expressed according to the following Equation (4):

(4) $$Protein\;Solubility\left(\%\right)=\frac{P_{soluble}}{P_{tot}}\cdot 100$$

where $P_{soluble}$ corresponds to the mass of soluble proteins (g) in the (-P) or (-S) fractions, while $P_{tot}$ represents the protein mass (g) present in the respective fractions.

### 2.6. Phycobiliprotein Determination in the Soluble Fraction

Phycobiliproteins determination in the freeze-dried supernatants obtained after isoelectric precipitation was carried out following the method described by Wang et al. [19], with minor modifications. Each supernatant fraction sample was solubilized at 0.5% *w*/*v* in PBS (0.1 M, pH 7) and stirred at room temperature for 1 h at 20 °C. The absorbance of the resulting solution was measured using a UV/Vis spectrophotometer (VWR, V-3000PC, VWR International, Milano, Italy) at 280, 562, 620 and 652 nm. The concentrations of C–phycocyanin (C−PC), allophycocyanin (APC) and phycoerythrin (PE) were calculated using Equations (5)–(7) [29]:

(5) $$C-PC\left(\frac{mg}{mL}\right)=\frac{A_{620}-0.474A_{652}}{5.34}$$

(6) $$APC\left(\frac{mg}{mL}\right)=\frac{A_{652}-0.208A_{620}}{5.09}$$

(7) $$PE\left(\frac{mg}{mL}\right)=\frac{A_{562}-2.41\left(C-PC\right)-0.849\left(APC\right)}{9.62}$$

The total phycobiliproteins content $\left({PBP}_{s}\right),$ expressed as mg per g of freeze-dried supernatant, was calculated using Equation (8):

(8) $${PBP}_{s}\left(\frac{mg}{g}\right)=\frac{\left[\left(C-PC\right)+\left(APC\right)+\left(PE\right)\right]\;\cdot \;V}{m}$$

where V is the volume of the supernatant used (mL), and m is the mass of the supernatant (g).

The C−PC extract purity index $\left(EP\right)$, which provides an indication of protein purity, was calculated according to Wang et al. [19], using Equation (9):

(9) $$EP=\frac{A_{620}}{A_{280}}$$

where A refers to the absorbance value recorded at 620 nm and 280 nm, respectively.

### 2.7. Emulsifying Properties

The emulsifying activity (EAI) and stability (ESI) index of the ALK-P and HPH-P were determined according to Purdi et al. [22], with slight modifications. A 0.1% *w*/*v* precipitate solution was prepared in PBS (0.1 M) and at pH values equal to 3, 5, 7, 9 and 11. The solutions were mixed with palm oil at a 1:4 ratio. Emulsions where homogenized (10,000 rpm, 1 min) using a T25 Ultra-Turrax (IKA, Staufen Im Breisgau, Germany). Then, 50 µL of the homogenised solution was diluted in 5 mL of a 0.1% *w*/*v* solution of sodium dodecyl sulfate (SDS). The absorbance ($A_{0}$) of the emulsions was determined at 500 nm using a UV/Vis spectrophotometer (VWR, V-3000PC, VWR International, Milano, Italy) with 0.1% SDS solution as the blank control. After 10 min, the absorbance ($A_{t}$) was measured again. EAI and ESI were calculated as follows using Equations (10) and (11):

(10) $$EAI\left(\frac{m^{2}}{g}\right)=\frac{2.303\;\cdot \;2\;\cdot \;A_{0}\;\cdot \;DF}{\varepsilon \theta C\;\cdot \;10^{4}}$$

(11) $$ESI\left(\min\right)=\frac{A_{0}}{A_{0}-A_{t}}\;\cdot \;∆t$$

where DF is the dilution factor (101); $A_{0}$ and $A_{t}$ are the absorbances of the diluted emulsified solutions at 0 and 10 min, respectively; ε is the optical path (1 cm); θ is the volume fraction of the oil phase (0.25); and C is the concentration of the protein in the precipitate solution before emulsification and ∆t=10 min.

### 2.8. Differential Scanning Calorimetry for the Thermal Analysis of Spirulina Protein Extracts

The thermal properties of the Spirulina protein extracts were analyzed using a Differential Scanning Calorimeter (DSC Q2000, TA Instruments, New Castle, DE, USA) according to Faieta et al. [30], with slight modifications. Approximately, 10–15 mg of each dried sample was accurately weighted into standard aluminium pans, which were then hermetically sealed. A sealed empty pan was used as the reference. The analysis was performed under an inert nitrogen atmosphere at a flow rate of 50 mL/min. The instrument was calibrated for both temperature and enthalpy using pure indium. The samples were heated from 20 to 110 °C at a constant rate of 5 °C/min. Thermographs were analyzed using the Universal Analysis software (TA Instruments). The onset temperature (To), peak denaturation temperature (Tp) and denaturation enthalpy (ΔH) were determined. ΔH values were normalized to the total protein content of each sample, as described by Shrestha et al. [31], using the following equation:

(12) $${∆H}_{P}\left(\frac{J}{g_{P_{tot}}}\right)=\frac{∆H}{P_{tot}}$$

where ${∆H}_{P}$ represents the denaturation enthalpy per gram of total proteins, ∆H is the enthalpy measured by DSC and $P_{tot}$ is the total protein mass (g) in the precipitate (-P) or supernatant (-S) fractions.

### 2.9. Rheological Measurements

The rheological behaviour of precipitates was evaluated following the procedure described by Shkolnikov Lozober et al. [25], with minor modifications. Suspensions of ALK-P (18% *w*/*v*) and HPH-P (15% *w*/*v*) were prepared using PBS (0.1 M, pH 7) and gently stirred until complete dispersion. These concentrations were chosen based on the least gelation concentration (LGC) determined in our previous work [32]. All measurements were performed using a rheometer (MCR 102e, Anton Paar GmbH, Rivoli, Italy) equipped with a parallel plate geometry (PP25, diameter 25 mm) and a Peltier heating and cooling system. To minimize water evaporation during heating, silicone oil was applied around the sample. The linear viscoelastic region (LVR) was identified by amplitude sweep tests performed at 1 Hz over a strain range of 0.001–100%. A constant strain of 0.01%, within the LVR, was selected for all subsequent tests. After gel formation on the rheometer, the LVR was reassessed and yielded at the same limit; therefore, a strain of 0.01% was used for all gel measurements. Temperature sweep was undertaken by heating protein dispersion from 20 °C to 90 °C at a rate of 3 °C/min, followed by an isothermal hold at 90 °C for 20 min and subsequent cooling to 20 °C at 5 °C/min. After cooling, the samples were equilibrated for 5 min at 20 °C. Throughout the entire heating–cooling cycle, storage modulus (G′) and loss modulus (G″) were continuously recorded using RheoCompass™ (version 1.32) software (Anton Paar GmbH). Finally, frequency sweep tests were performed within the angular frequency of 0.1–100 rad/s at 20 °C, maintaining the strain within the LVR.

### 2.10. Statistical Analysis

All the experiments were carried out in triplicate, and the data were reported as mean values ± the standard deviation. One-way analysis of variance (ANOVA) and pairwise comparisons among samples were conducted using Tukey’s test to determine statistically significant differences at a significance level of *p* < 0.05. The data were analyzed using JMP statistical (version 18) software (SAS Institute Inc., Cary, NC, USA).

## 3. Results and Discussion

### 3.1. Impact of Alkaline and HPH on the Cellular Microstructure of Spirulina Suspensions

**Figure 2:**
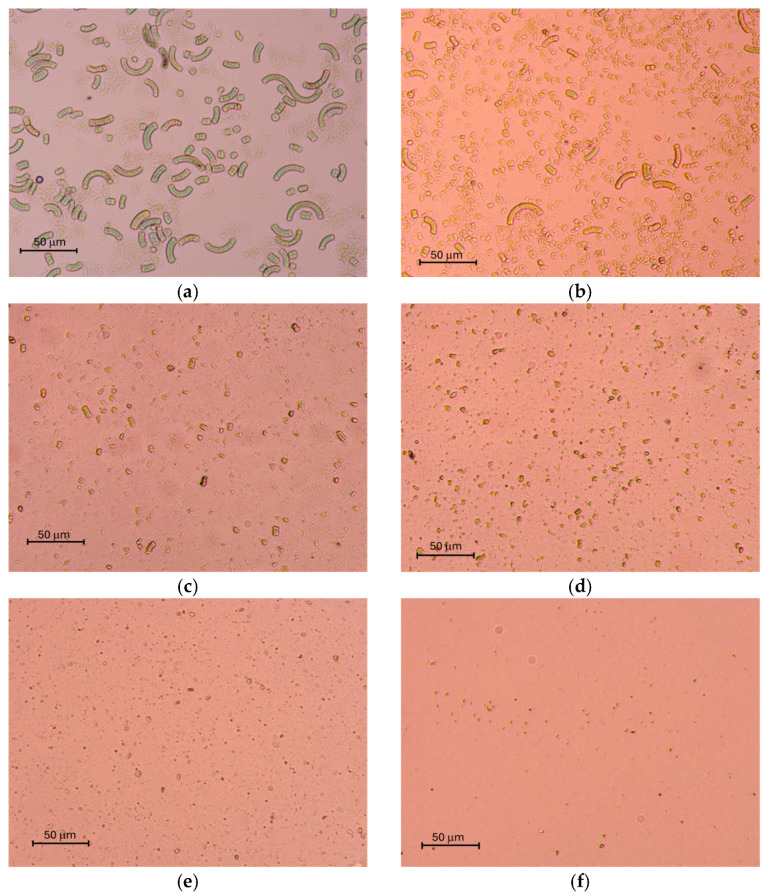
Optical microscopy images (100× magnification) of Spirulina suspensions after different extraction treatments. (**a**) Untreated SB control; (**b**) alkaline extraction; (**c**–**f**); HPH at 20, 50, 80 and 100 MPa, respectively.

Optical microscopy images of Spirulina suspensions, before and after alkaline and HPH extraction, are shown in Figure 2. In the untreated biomass (Figure 2a), the native helical, filamentous trichomes morphology of Spirulina is not readily visible, probably due to the drying process used to produce Spirulina in powder [24]. Following alkaline extraction (Figure 2b), partial degradation of the trichomes was observed in the Spirulina suspension, due to alkaline pH-induced weakening of the peptidoglycan layer [21]. The alkaline pH employed during alkaline extraction (typically >10) promotes partial degradation of the peptidoglycan layer, facilitating trichome disaggregation. This effect is attributed to the hydrolysis of peptide cross-links and increased membrane permeability, ultimately weakening the filamentous structure of Spirulina [33,34,35]. The application of HPH (Figure 2c–f) induced progressive trichomes disruption in a pressure-dependent manner. At 20 MPa, the spiral filaments were shortened and fragmented into smaller segments. At 50 MPa, morphological degradation becomes more pronounced, leading to the complete loss of filamentous structures and the appearance of several submicron debris at 80 MPa. At 100 MPa, extensive destruction was observed. These results are consistent with previous studies reporting the mechanical fragility of Spirulina, attributed to its low ability to resist shear stresses generated already at moderate pressures [24,36].

### 3.2. Protein Extraction Efficiency

The total protein extraction yield, defined as the sum of proteins recovered in the precipitate and in the supernatant, reflects the overall recovery of proteins initially present in the Spirulina biomass. For the HPH-treated samples, the total protein extraction yield increased up to 50 MPa and then reached a plateau. The alkaline method and HPH at 20 MPa exhibited statistically comparable values, as shown in Table 2. However, the protein content of the two fractions reveals a clear pressure-dependent redistribution; as pressure increases, the precipitate shows a progressive decrease in protein content, whereas the supernatant exhibits an increase. The observed pressure-dependent redistribution indicates enhanced cell disruption and protein solubilization driven by shear and partial unfolding at higher mechanical stresses [23]. This behaviour is further supported by our previous study [32], where the protein yield of the precipitate obtained by alkaline extraction was the lowest compared to all HPH-treated samples. Moreover, at moderate pressures (20 and 50 MPa), an increase in protein yield was observed, followed by a decrease at higher pressures. Furthermore, the evaluation of the supernatant protein yield in the present study provides additional insights. The supernatant protein yield shows the opposite behaviour at the same pressures, confirming that homogenization enhances protein recovery in the soluble fraction. This indicates that HPH improves protein recovery relative to alkaline extraction and drives a pressure-dependent redistribution from the insoluble to the soluble fraction. For the HPH extraction, our total protein extraction yield was lower than the 96% reported by Elain et al. [37] at 50 and 100 MPa. However, their extraction method involved seven homogenization cycles, which could enhance the cell disruption and may explain their higher recovery. In contrast, under alkaline extraction at pH 10, our results were higher than those of Purdi et al. [22], who reported a 12.5% protein yield and 63% protein content. Similarly, the protein content of our precipitated fraction reached 77%, higher than the 73% reported by Shkolnikov Lozober et al. [25], under similar extraction conditions. For the supernatant fraction, to the best of our knowledge, no studies report protein recovery suitable for a direct comparison. Therefore, the present study provides a significant contribution by directly evaluating both fractions, offering a more comprehensive understanding of the extraction process and the pressure-dependent redistribution of proteins.

**Figure 3:**
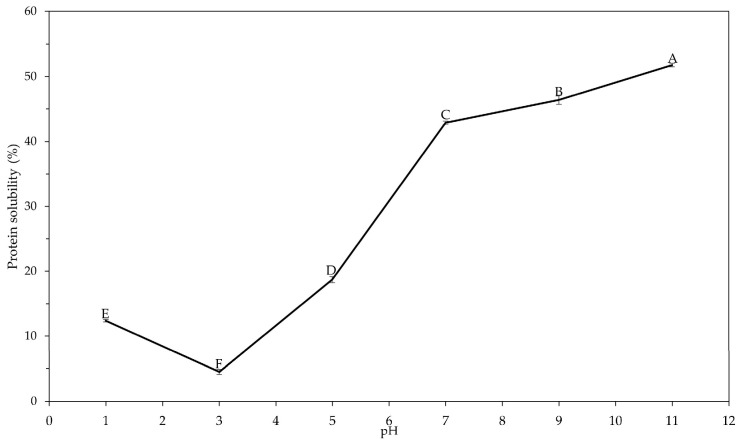
Protein solubility (%) of ALK-P at different pH values. Different letters (A–F) indicate statistically significant differences between means (*p* < 0.05).

Moreover, protein solubility was further evaluated to determine whether the pressure-dependent redistribution between the precipitate and supernatant affected their functional behaviour. Protein solubility is one of the most important functional properties of proteins, as it influences several other functional properties such as emulsifying, foaming and gelling properties [26]. It is influenced by the amino acid composition and surface charge of the protein, as well as by processing conditions such as pH and extraction methods [38]. The solubility of the ALK-P exhibited a typical U-shaped profile as a function of pH, with a minimum at pH 3 and a significant increase (*p* < 0.05) under alkaline conditions (Figure 3), consistent with previous studies [38,39]. This profile is in line with Benelhadj et al. [40], who reported a minimum of 6.2% at pH 3 followed by a gradual increase at higher pH values. This behaviour reflects the reduction in net charge near the isoelectric point, which promotes aggregations, and the enhanced protein–water interactions at alkaline pH due to the deprotonation of acidic residues (Glu, Asp) [11,41]. The present HPH 80-P value (47.73 ± 0.60%) is consistent with the pressure-dependent trend observed in our previous work [32], where solubility increases at moderate pressures before decreasing at higher intensity. This pattern indicates that pressures in the range of 20–50 MPa promote partial unfolding and strengthen protein–solvent interactions, whereas higher pressures expose hydrophobic regions, leading to aggregation and a consequent decrease in solubility [42]. Similar pressure-dependent effects have been reported for plant proteins, with chickpea isolates showing maximum solubility close to 90 MPa, followed by a subsequent reduction at higher pressures [43].

**Figure 4:**
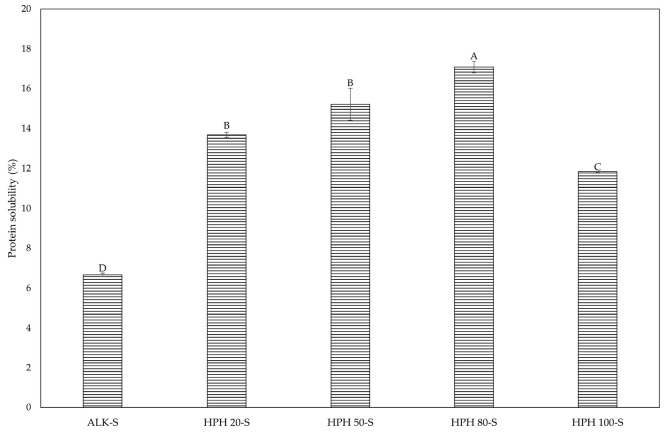
Protein solubility (%) at pH 7 of Spirulina supernatant fractions obtained by alkaline treatment (ALK-S) and HPH at different pressures (20, 50, 80 and 100 MPa). Different letters indicate statistically significant differences among samples (*p* < 0.05).

The solubility of the supernatant fractions was strongly influenced by the extraction method and applied pressure. While alkaline extraction produced the lowest solubility, HPH improved protein solubilization, with the highest levels observed at 80 MPa (Figure 4). The pressure-dependent trend observed is consistent with our previous findings on the precipitates [32], where solubility increased from 39.05% at 20 MPa to a maximum of 50.74% at 50 MPa, before declining to 31.60% at 100 MPa. The present value at 80 MPa (47.73%) confirms this trend, being lower than the maximum at 50 MPa and followed by a further decrease at 100 MPa. However, supernatant solubility reached the highest value equal to 17.3% at 80 MPa and then decreased to 12% at 100 MPa. These results are in contrast with expectations, as the proteins fraction present in the aqueous phase, mainly phycocyanin’s, as discussed in the next paragraph, should exhibit high solubility [44]. A possible explanation for this result could be the analytical method employed. Protein solubility was quantified using the Bradford assay, which relies primarily on Coomassie Brilliant Blue G-250 dye binding to basic and aromatic amino acid residues, especially arginine [45,46]. In contrast, phycobiliprotein-rich supernatants are characterized by a higher content of acidic residues (Asp, Glu) and a lower amount of basic residues (Arg, Lys, His), leading to underestimation [47,48].

### 3.3. Proteins Characterization of Fractions Obtained by HPH and Alkaline Methods

The analysis of the supernatant fractions revealed a clear effect of the extraction method and applied pressure on the distribution of phycobiliproteins (PBPs), as shown in Table 3. C–phycocyanin (C–PC) was the predominant component, followed by phycoerythrin (PE), whereas allophycocyanin (APC) was only detected in trace amounts under ALK and 80 MPa. PBPs were higher in HPH-treated samples compared to the alkaline extraction, suggesting that mechanical disruption promotes the release of pigmented proteins into the soluble fraction. The maximum recovery was observed at intermediate pressures (50 and 80 MPa), followed by a partial decrease at the highest level, which is consistent with the structural sensitivity of PBPs to intense mechanical stress [21]. When compared to literature values, the percentage of C–PC obtained in this study falls within the typical range reported for Spirulina biomass, where C–PC usually accounts for 10–20% of total proteins [21,29,49]. It should be noted, however, that the present results refer exclusively to the supernatant fractions. Since PBPs in the insoluble precipitates were not quantified, the actual PBPs content could be underestimated. C–PC not only provides the characteristic blue colour of increasing interest in clean-label food formulations but also exhibits antioxidant, anti-inflammatory and immunomodulatory activities, supporting its inclusion in functional foods and nutraceuticals [44]. In this regard, HPH emerges not only as an effective method for PBPs recovery but also a method for modulating their distribution as a function of pressure, with direct implications for both yield and techno-functional quality. To the best of the authors’ knowledge, no previous study employed HPH for the extraction of PBPs from Spirulina and investigated the effect of pressure on the recovery and portioning of PBPs and C–phycocyanin. Published articles on Spirulina used aqueous or buffered extraction, coupled with sonication or enzymatic lysis [18,50]. In terms of the purity index (EP), a value above 0.7 is generally associated with food-grade purity, whereas values greater than 1.0–1.5 are suitable for analytical or reagent-grade applications [51]. The EP values of the extracts obtained at the highest pressures were statistically higher (*p* < 0.05) than the alkaline extraction, reaching its maximum at 100 MPa. HPH was shown to be effective for the extraction of phycobiliproteins, particularly C–phycocyanin with high purity.

### 3.4. Water Holding Capacity and Emulsifying and Foaming Properties

**Figure 5:**
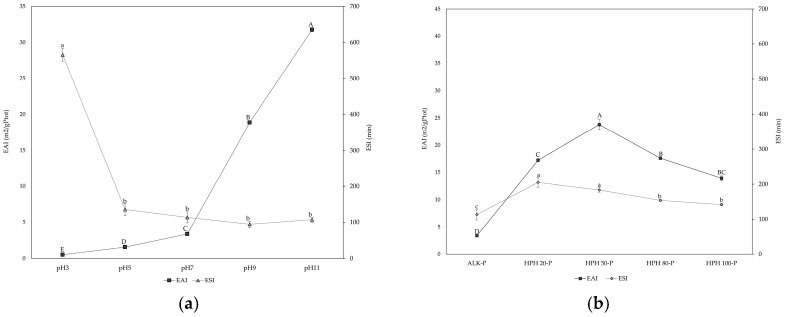
Emulsifying Activity Index (m^2^/gPtot) and Emulsion Stability Index (min) of protein extracts from Spirulina. (**a**) Effect of pH (3, 5, 7, 9 and 11) on the emulsifying properties of the ALK-P; (**b**) Comparison between ALK-P at pH 7 and samples extracted by HPH at 20, 50, 80 and 100 MPa. Different letters indicate statistically significant differences among samples (*p* < 0.05) for each parameter (EAI: uppercase letters A–E; ESI: lowercase letters a–c).

The EAI provides an estimate of how effectively proteins can adsorb onto the oil–water interface during emulsion formation, while the ESI reflects their ability to preserve the structural integrity of the emulsion over time [43,52]. The EAI of the ALK-P extract (Figure 5a) showed a strong dependence on pH, with values increasing progressively from acidic to alkaline conditions. The lowest EAI was observed at pH 3, while the highest was at pH 11. These results suggest that alkaline conditions promote protein solubilization, unfolding and the exposure of hydrophobic and charged groups, improving their ability to adsorb and stabilize oil–water interfaces [53]. Interestingly, the ESI exhibited the opposite trend, being highest at pH 3, where the EAI was the lowest. Since the minimum protein solubility at acidic pH may hinder the initial emulsification process [53]. The EAI obtained via HPH was significantly higher (*p* < 0.05) than that of ALK-P, showing a clear pressure-dependent trend, as shown in Figure 5b. EAI increased progressively up to 50 MPa, where it reached the maximum, before declining at higher pressures (80 and 100 MPa). This behaviour is consistent with findings on legume and microalgal proteins [43], where moderate homogenization pressures promoted enhanced emulsifying capacity, whereas excessive mechanical stress led to a loss of interfacial functionality. The improvement observed at intermediate pressures can be attributed to the partial unfolding and dissociation of protein structures, which increase conformational flexibility and expose hydrophobic residues otherwise buried in the protein matrix [54]. These residues facilitate rapid adsorption at the oil–water interface and reduce interfacial tension, improving droplet dispersion [55]. However, above 50 MPa, the reduction in EAI and particularly in ESI indicates that excessive structural disruption inhibits the formation of a cohesive interfacial film, despite the higher solubility of proteins treated at 100 MPa. This pressure-driven shift in the hydrophilic/hydrophobic balance is also evident in the changes in techno-functional properties observed in our previous study [32]. In fact, the progressive exposure of hydrophobic groups following HPH explains the increase in oil-holding capacity (OHC) and foaming capacity (FC) at moderate pressures, whereas the concomitant decrease in water-holding capacity (WHC) reflects the reduced hydration ability of proteins once hydrophobic interactions prevail. Such changes confirm that the unfolding and rearrangement of protein structures improve their interfacial reactivity with oil solvents and air–water interfaces. At high pressures (e.g., 100 MPa), the loss of OHC and the decline in foaming stability (FS), in parallel with the drop in ESI, suggest that the disruption of the protein tertiary and quaternary structure exceeds the threshold of functional benefit, leading to aggregation and reduced interfacial film integrity [42,52]. Overall, the combined interpretation of emulsifying, foaming and water/oil-holding properties indicates that HPH induces a balance between beneficial structural flexibility and detrimental over-denaturation, with optimal functionality achieved at a moderate pressure of 50 MPa.

### 3.5. Thermal Stability and Denaturation Behaviour of Spirulina Extracts

The thermal properties of proteins indicate their stability during heating treatment and the structural changes occurring during the transition from a native to denatured state [31]. Among the precipitate fractions, HPH 50-P exhibited the highest onset (To) and peak (Tp) denaturation temperatures and the maximum enthalpy change (ΔH_P_), as reported in Table 4. These results indicate a more ordered and thermally stable protein structure promoted by moderate pressure, which facilitates controlled molecular rearrangements [23].

In contrast, higher pressures (80–100 MPa) caused a marked reduction in the thermal parameters, particularly in HPH 80-P, which displayed the lowest To and Tp values. This suggests partial denaturation and aggregation arising from the exposure of hydrophobic regions, leading to reduced structural order and less energetic thermal transitions. Similar trends were reported by Kelany and Yemiş [56], who demonstrated that excessive pressure disrupts proteins conformation.

Compared with HPH-treated samples, ALK-P showed lower To and Tp than HPH 50-P but higher To and Tp than those of HPH 80-P. Moreover, the significantly lower ΔH_P_ of ALK-P compared with HPH 50-P indicates a less ordered and more easily unfolded protein network, thereby confirming the more structured nature of the 50 MPa sample.

For the supernatants, ALK-S exhibited the highest ΔH, suggesting the preservation of native proteins structures in the aqueous phase due to the absence of mechanical disruption [39]. Interestingly, all HPH-S samples displayed an endothermic peak near 49 °C, consistent with the thermal behavior of phycoerythrin and C–phycocyanin, which undergo structural unfolding and chromophore destabilization between 45 and 50 °C [25,57,58]. HPH-treated supernatants showed lower ΔH values, indicating that high mechanical stress partially disrupted protein conformations, thereby reducing the energy required for denaturation [59].

Similar findings were reported by Liang et al. [59], who investigated the effects of high-pressure homogenization on soybean protein concentrates. They observed that, although HPH enhanced protein solubility, it simultaneously reduced denaturation enthalpy (ΔH_P_) due to the partial unfolding and aggregation induced by mechanical forces. Likewise, Ricci et al. [60] highlighted that ΔH is more closely associated with the proportion of structurally native proteins than with the total protein content. These findings support the interpretation that the high ΔH observed for ALK-S reflects a greater degree of conformational integrity, despite its lower overall protein concentration.

### 3.6. Rheological Properties of Spirulina Protein Extracts: Flow and Viscoelastic Properties

**Figure 6:**
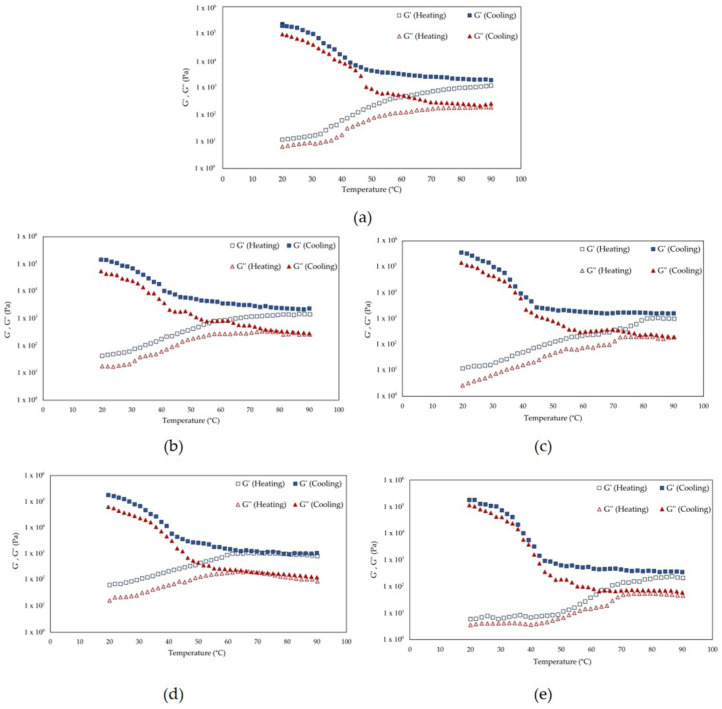
Temperature-dependent viscoelastic moduli G′ (storage modulus) and G″ (loss modulus) of Spirulina protein extracts during heating (solid lines) and cooling (dashed lines) cycles. The samples include (**a**) ALK-P and those obtained by HPH at (**b**) 20 MPa, (**c**) 50 MPa, (**d**) 80 MPa and (**e**) 100 MPa.

The temperature sweep profiles in Figure 6 describe the temperature-dependent viscoelastic behaviour of the precipitate obtained by alkaline and HPH methods. All samples showed G′ values slightly higher than G″ throughout both heating and cooling, with no crossover observed within the investigated temperature range. This elastic-dominant profile suggests the formation of weakly structured protein networks that do not undergo a typical sol-gel transition over the investigated temperature range, in line with previous findings on pulse and microalgal proteins [61,62,63]. The ALK-P sample (Figure 6a) exhibited during heating a progressive increase in G’―particularly between 30 and 60 °C―suggesting partial denaturation and disruption of protein–protein interactions [62]. During cooling, G′ reached higher values than the initial ones, probably due to the formation of a new reorganised network. This trend can be attributed to hydrophobic interaction and the entanglement of partially unfolded chains [31]. Similar profiles have been described for pea proteins, where heat treatment led to the exposure of hydrophobic regions and subsequent reaggregation during cooling [61]. The 50 MPa treatment showed the highest viscoelastic moduli among all precipitate fractions, while HPH 100-P showed the lowest G′. This trend is consistent with the observations of Shkolnikov Lozober et al. [25], who reported that HPH at 50 MPa improves protein solubility and surface hydrophobicity and thus promotes intermolecular interactions. These results highlight that moderate homogenization pressure improves the unfolding and exposure of hydrophobic residues, promoting protein–protein interactions and the formation of a more elastic network without causing extensive structural breakage. At higher pressures, however, excessive shear forces and cavitation could disrupt the protein structure and weaken the network as intense mechanical stresses during HPH.

**Figure 7:**
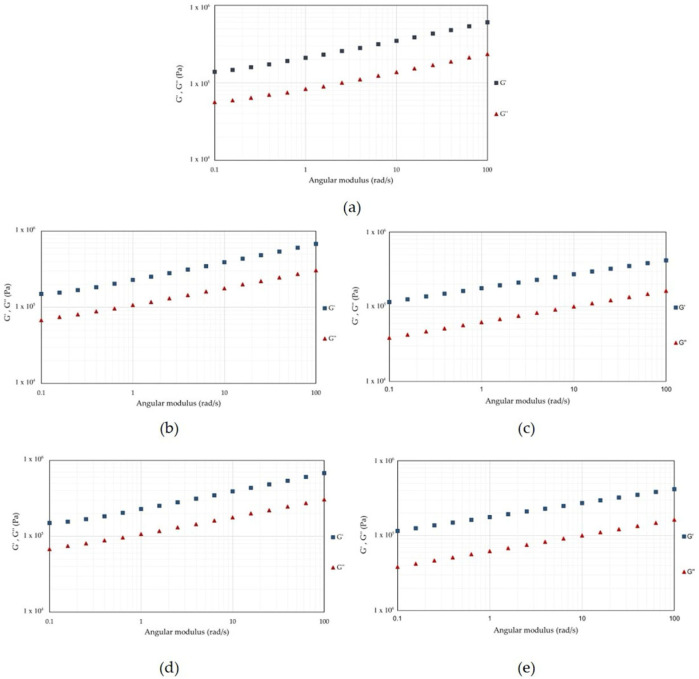
Frequency sweep profiles of Spirulina protein extracts showing the storage modulus (G′, squares) and loss modulus (G″, triangles) as a function of angular frequency (0.1–100 rad/s). Samples were obtained by (**a**) ALK and by HPH at (**b**) 20 MPa, (**c**) 50 MPa, (**d**) 80 MPa and (**e**) 100 MPa.

The frequency sweep analysis (Figure 7) confirmed the elastic behaviour in all samples as G′ over G″ across the entire angular frequency range investigated. This indicates that all systems behaved as viscoelastic solids, as previously reported for Spirulina and other microalgae or pulse protein systems [63,64], and showed a frequency dependence, which is characteristic of protein gel systems exhibiting weak-gel behaviour [62]. Compared to ALK-P, HPH treatments at moderate pressures (20 and 50 MPa) resulted in a more pronounced difference between G′ and G″ than at 80 and 100 MPa. This trend suggests that intermediate pressure promotes partial protein unfolding, leading to the exposure of hydrophobic regions and reactive groups that enhance intermolecular interactions (hydrophobic, electrostatic and hydrogen bonding), thereby supporting the formation of a more cohesive three-dimensional network [23,25].

The loss factor (tan δ) showed in Table 5, ranging between 0.33 and 0.45 across all samples, confirms a weak-gel structure (tan δ > 0.1) [62,65]. The highest value was observed at 20 MPa, suggesting a more dissipative network with increased viscous contribution. At 50 MPa, tan δ was comparable to the alkaline extract, whereas as the pressure increased to 80 and 100 MPa, tan δ progressively decreased, reaching the minimum at 100 MPa. This could suggest that the system became more elastic. Although lower values suggest a more elastic response, this trend is probably a result of the formation of compact protein aggregates, which increase the rigidity of the network but may reduce its ability to reorganize structurally under deformation. Similar results have been reported in other protein systems exposed to high mechanical stress or pressure where aggregation phenomena led to the formation of networks that appeared more elastic but exhibited a limited functionality [16,62,63].

**Table 5:** Loss factor (tan δ) values of Spirulina protein precipitate obtained through alkaline and HPH extraction conditions.

Sample	Loss Factor
ALK -P	0.40 ± 0.01 ^AB^
HPH 20-P	0.45 ± 0.03 ^A^
HPH 50-P	0.38 ± 0.02 ^AB^
HPH 80-P	0.38 ± 0.05 ^BC^
HPH 100-P	0.33 ± 0.04 ^C^

Different letters (A–C) within each column indicate statistically significant differences among samples (*p* < 0.05).

## 4. Conclusions

This study compared alkaline extraction and HPH at 20, 50, 80 and 100 MPa to assess their effects on the functional properties of Spirulina proteins. HPH induces a clear pressure-dependent redistribution of proteins between precipitate and supernatant fractions. At 50 MPa, the precipitate fraction exhibited the highest protein yield and content. The supernatant fractions were enriched in phycobiliproteins―particularly C–phycocyanin―with higher purity than those obtained by alkaline extraction. The maximum recovery of phycobiliproteins was achieved at intermediate pressures (50–80 MPa), whereas treatment at 100 MPa led to partial structural degradation and lower extraction efficiency. The HPH 50-P also exhibited improved emulsifying and foaming capacities and enhanced oil-holding ability compared with the alkaline extract. Thermal analysis showed a maximum at 50 MPa, with higher denaturation temperature and enthalpy, in line with a more ordered and thermally stable structure. Consistently, rheological analysis confirmed a weak gel-like behaviour for all samples, with the lowest viscoelastic moduli recorded at 80 and 100 MPa, indicating that excessive mechanical stress disrupts the protein network. Overall, HPH proved to be an effective and sustainable alternative to conventional Spirulina protein extraction. Although alkaline extraction is simpler and more cost-effective, it is limited by reagent use and batch processing. In contrast, HPH extraction, despite higher initial costs, enables continuous operation, reduced alkali consumption and improved yield and functionality, representing a more economically and environmentally sustainable approach while allowing for the modulation of proteins structures and functionalities through pressure control. These findings highlight HPH as a promising technology for producing high-quality functional proteins and natural pigments suitable for use in next-generation sustainable food formulations.

## Figures and Tables

**Table 1:** Sample codes obtained by alkaline and high-pressure homogenization extraction.

Extraction Method	Pressure (MPa)	Precipitate Fraction (-P)	Supernatant Fraction (-S)
Alkaline (ALK)		ALK-P	ALK-S
High-Pressure Homogenization (HPH)	20	HPH 20-P	HPH 20-S
	50	HPH 50-P	HPH 50-S
	80	HPH 80-P	HPH 80-S
	100	HPH 100-P	HPH 100-S

**Table 2:** Total protein extraction yield and protein content in precipitate and supernatant for extract obtained by alkaline extraction and HPH at 20–100 MPa.

Method	Total Protein Extraction Yield g_Ptot_/100g_PSB_	Precipitate		Supernatant	
		Protein Content g_Ptot_/100g_-P_	Protein Yield * g_Ptot_/100g_PSB_	Protein Content g_Ptot_/100g_-P_	Protein Yield * g_Ptot_/100g_PSB_
ALK	56.58 ± 0.40 ^C^	77.32 ± 0.43 ^A^	31.21 ± 0.83 ^D^	21.14 ± 1.32 ^D^	8.03 ± 0.16 ^C^
HPH 20	60.53 ± 1.43 ^C^	67.87 ± 0.40 ^B^	55.82 ± 1.24 ^C^	30.03 ± 0.28 ^C^	4.71 ± 0.19 ^D^
HPH 50	72.35 ± 1.15 ^B^	69.00 ± 0.18 ^B^	68.02 ± 0.92 ^A^	30.54 ± 0.46 ^C^	4.34 ± 0.24 ^D^
HPH 80	73.62 ± 1.51 ^AB^	58.96 ± 0.11 ^C^	63.32 ± 1.20 ^B^	38.07 ± 0.94 ^B^	10.30 ± 0.31 ^B^
HPH 100	77.79 ± 0.74 ^A^	49.06 ± 0.13 ^D^	58.42 ± 0.30 ^C^	50.06 ± 0.84 ^A^	19.49 ± 0.73 ^A^

Data are mean ± SD; different letters within the column indicate a significant difference (*p* < 0.05) among samples. * Precipitate protein yield values adapted from Muccio et al. [32].

**Table 3:** Total phycobiliproteins (PBPs), C–phycocyanin (C–PC) content and purity index (EP) of the freeze-dried supernatants obtained from alkaline and HPH extraction methods.

Sample	PBPs g_PBP_/100g_Ptot-S_	C-PC g_C-PC_/100g_Ptot-S_	A-PC g_APC_/100g_Ptot-S_	PE g_PE_/100g_Ptot-S_	EP -
ALK	3.45 ± 0.09 ^E^	2.40 ± 0.03 ^E^	0.36 ± 0.04 ^A^	0.69 ± 0.02 ^A^	0.27 ± 0.10 ^B^
HPH 20	7.07 ± 0.00 ^D^	6.65 ± 0.00 ^D^	0.00 ± 0.00 ^B^	0.04 ± 0.00 ^C^	0.53 ± 0.00 ^B^
HPH 50	10.42 ± 0.00 ^B^	9.61 ± 0.00 ^A^	0.00 ± 0.00 ^B^	0.08 ± 0.00 ^B^	0.91 ± 0.14 ^A^
HPH 80	10.62 ± 0.00 ^A^	9.23 ± 0.00 ^B^	0.07 ± 0.00 ^B^	0.07 ± 0.00 ^BC^	0.96 ± 0.02 ^A^
HPH 100	8.12 ± 0.02 ^C^	7.62 ± 0.02 ^C^	0.00 ± 0.00 ^B^	0.05 ± 0.00 ^BC^	1.04 ± 0.00 ^A^

Data are presented as the mean ± standard deviation (*n* = 3). Different letters (A–E) in the same column indicate significant (*p* < 0.05) differences between treatments.

**Table 4:** Thermal parameters (To, Tp, ΔH_P_) of protein fractions from precipitate (-P) and supernatant (-S) after alkaline and HPH extraction (20–100 MPa).

Sample	T_O_ °C	T_P_ °C	ΔH_P_ J/g_Ptot_
ALK-P	48.56 ± 0.86 ^B^	51.57 ± 0.52 ^B^	2.75 ± 0.11 ^B^
HPH 20-P	44.49 ± 0.71 ^C^	49.37 ± 0.00 ^B^	1.17 ± 0.30 ^B^
HPH 50-P	53.53 ± 0.86 ^A^	58.70± 0.56 ^A^	11.96± 0.20 ^A^
HPH 80-P	38.54 ± 0.40 ^D^	45.50 ± 0.42 ^C^	2.61 ± 0.70 ^B^
HPH 100-P	47.22 ± 0.85 ^BC^	50.42 ± 1.24 ^B^	8.79 ± 1.61 ^A^
ALK-S	41.80 ± 0.59 ^C^	41.19 ± 0.02 ^B^	19.27 ± 0.10 ^A^
HPH 20-S	47.81 ± 0.97 ^AB^	50.18± 1.56 ^A^	4.22 ± 1.31 ^B^
HPH 50-S	46.10 ± 0.28 ^B^	47.63 ± 0.05 ^A^	5.36 ± 1.16 ^B^
HPH 80-S	48.64 ± 0.54 ^A^	50.14 ± 0.48 ^A^	5.11 ± 0.46 ^B^
HPH 100-S	39.85 ± 0.21 ^C^	47.72 ± 0.11 ^A^	8.53 ± 1.89 ^B^

Different letters within each column indicate statistically significant differences among samples (*p* < 0.05).

## Data Availability

The original contributions presented in this study are included in the article. Further inquiries can be directed to the corresponding author.
